# Infektionsprävention im OP: praxisorientierte Empfehlungen für AnästhesistInnen

**DOI:** 10.1007/s00101-022-01239-z

**Published:** 2022-11-30

**Authors:** Maximilian Schnetzinger, Ojan Assadian, Klaus Markstaller, Klaus Ulrich Klein

**Affiliations:** 1 Univ.-Klinik für Anästhesie, Allgemeine Intensivmedizin und Schmerztherapie, Medizinische Universität Wien, Währinger Gürtel 18–20, 1190 Wien, Österreich; 2 Ärztliche Direktion, Landesklinikum Wiener Neustadt, Corvinusring 3–5, 2700 Wiener Neustadt, Österreich

**Keywords:** Perioperative Hygiene, Perioperative Wundinfektion, Hygieneempfehlung, Leitlinie, Infektionsrisiko, Perioperative hygiene, Surgical site infections, Hygiene recommendations, Guidelines, Risc of infection

## Abstract

Der vorliegende Artikel soll klinisch tätigen AnästhesistInnen einen praxisorientierten Überblick zu ausgewählten, wichtigen Empfehlungen der Hygiene im OP-Bereich vermitteln. Die Inhalte des Artikels basieren auf den Leitlinien oder Empfehlungen der World Health Organisation (WHO), der Arbeitsgemeinschaft der Wissenschaftlichen Medizinischen Fachgesellschaften (AWMF) sowie der Kommission für Krankenhaushygiene und Infektionsprävention (KRINKO) am Robert Koch-Institut (RKI). Die Zielsetzung der Autoren ist es, den Hygienestandard in der täglichen Praxis zu optimieren, um die Zahl perioperativer Infektionen zu reduzieren.

## Abstract (en)

This article is intended to provide clinically working anesthesiologists with a practice-oriented overview of selected important current guidelines and recommendations pertaining to intraoperative prevention of infection. The contents of this article are based on the guidelines or recommendation of the World Health Organization (WHO) and the Association of the Scientific Medical Societies in Germany (AWMF) as well as the Commission for Hospital Hygiene and Infection Prevention (KRINKO) at the Robert Koch Institute (RKI). The authors’ objective is to foster and support the standard of infection prevention and control in the operating theater by optimizing the standard of hygiene in daily practice to reduce the number of perioperative infections.

## Einleitung

Die Sicherheit von Patientinnen und Patienten in der Anästhesie hat sich in den letzten Jahrzehnten drastisch erhöht, wozu auch das Bewusstsein für den Stellenwert der Infektionsprävention gehört. Dennoch ist bei 100 chirurgischen Eingriffen aktuell – je nach Art und Schweregrad des operativen Eingriffs (OP) und individueller Prädisposition der PatientInnen – mit einer Frequenz von 0,6–9,6 % mit einer postoperativen Wundinfektion („surgical side infection“, SSI) zu rechnen [1], wobei der anästhesieassoziierte Anteil von einigen Autoren auf bis zum 16 % geschätzt wird [2]. Der Fortschritt in der Prävention nosokomialer Infektionen im Gesundheitswesen ist daher von zentraler Bedeutung und stellt ein vorrangiges Ziel dar. Vor allem im OP-Bereich, in welchem PatientInnen aufgrund der OP und damit assoziierter Risiken besonders empfänglich für jede Form von Infektion sind, ist in besonderem Maß auf die Umsetzung von Hygienemaßnahmen zu achten.

Aus diesem Grund sind eine repetitive Diskussion sowie die Erstellung optimaler Behandlungskonzepte zur Reduktion von SSI von wesentlicher Bedeutung. Aktuelle Strategiekonzepte zur Vermeidung von SSI beinhalten Bündelmaßnahmen zur optimalen Vorbereitung der PatientInnen, Reinigung, Desinfektion, Be- und Entlüftung der Räumlichkeiten, Aufarbeitung und Sterilisierung von Material, Verwendung von sterilen Einmalprodukten, Durchführung der Anästhesie und Chirurgie, inklusive perioperativer Antibiotikaprophylaxe sowie einer frühen Identifikation und Therapie etwaiger SSI.

Die besondere Herausforderung an ein Krankenhaus und einzelne Abteilungen sowie Berufsgruppen ist die Schwierigkeit, die breit publizierte Evidenz in Form von Leitlinien und Bündelmaßnahmen bestmöglich umzusetzen. Denn aufgrund der hohen Komplexität der Evidenz existieren für jede Berufsgruppe zahlreiche Leitlinien und Vorgaben. Deshalb referenziert der vorliegende Artikel die Evidenz praktisch wichtiger Hygieneleitlinien und diskutiert ihre Umsetzung.

## Was ist bei der Durchführung der hygienischen Händedesinfektion zu beachten?

### Empfehlung.

Die hygienische Händedesinfektion (HD) soll, wie von der WHO empfohlen (*5 Moments For Hand Hygiene* [3]), durchgeführt werden. Die Durchführung einer HD mit einem alkoholbasierten Desinfektionsmittel (ABHR) ermöglicht eine Reduktion der Zahl von Erregern auf den Händen des Benutzers um 99,99–99,999 % – dies kann mit einer einzelnen Anwendung erreicht werden. Die Einwirkzeit, welche innerhalb der Präparate zwischen 15 und 30 s beträgt, ist zu beachten [4]. Die Autoren empfehlen, eine HD vor aseptischen Tätigkeiten (z. B. dem Legen eines ZVK, einer arteriellen Kanüle, dem Aufziehen von Medikamenten und dem Anstechen von Infusionen), nach dem Ausziehen von Einmalhandschuhen, bei Kontamination (z. B. oropharyngeales Sekret), vor dem Berühren *sauberer* Utensilien (z. B. Anästhesiewagen, Narkosegerät, Computertastatur) sowie beim Betreten oder Verlassen des OP.

### Diskussion.

Epidemiologische Studien deuten darauf hin, dass eine verbesserte intraoperative Händedesinfektion (HD) ein wesentlicher Bestandteil der intraoperativen Infektionsprävention im OP ist [5]. Die laut WHO geltenden Indikationen für die HD inkludieren die Zeitpunkte vor bzw. nach direktem Patientenkontakt, vor aseptischen Tätigkeiten, nach Kontakt mit Körperflüssigkeiten oder Schleimhäuten (beispielsweise nach erfolgter Intubation) und nach dem Berühren der unmittelbaren PatientInnenumgebung [6]. Wesentlich ist es in Erinnerung zu rufen, dass die von der WHO empfohlenen „5 Momente“ ursprünglich für die Pflege und Versorgung von bettlägerigen stationär versorgten PatientInnen im PatientInnenzimmer konzipiert wurden. Die WHO empfiehlt ausdrücklich, für spezielle Situationen, welche ein operatives Setting jedenfalls ist, eigene „Momente“ zu definieren. Momentan (Stand Juli 2020) liegen für typische anästhesiologische Handlungen allerdings keine eigenständigen Publikationen vor.

In verschiedenen Studien wurden sowohl die Gelegenheiten als auch die Compliance für das Durchführen einer HD gemäß WHO-Standards innerhalb des Anästhesiepersonals erfasst. In einer Beobachtungsstudie von Biddle et al. wurde die Durchführung der HD des Anästhesiepersonals von Experten registriert, die als Pflegepersonal getarnt an der Studie teilnahmen, um jegliche Einflussnahme auf die Durchführung der HD zu minimieren. Die Fehlerrate – im Sinne fehlender Compliance, die HD durchzuführen – aller TeilnehmerInnen betrug 82 % [7]. In selbiger Studie wurde beschrieben, dass während bestimmter kontaminationskritischer Situationen (z. B. großer Blutverlust, besonders anspruchsvolle Atemwegssicherung, Perioden von hoher Frequenz von Handgriffen am Patienten aufgrund von anästhesiologischen Notfallsituationen etc.) die WHO-Indikation für eine HD innerhalb einer Stunde bis zu 54-mal gestellt gewesen wäre, was mit einer Versagerquote von 83 % vergesellschaftet war.

Muñoz-Price et al. beschreiben in ihrer Studie, dass das Anästhesiepersonal innerhalb von 8 h nur durchschnittlich 13-mal eine HD durchführte. In einer Folgestudie stellten Muñoz-Price et al. fest, dass die Frequenz der durchgeführten HD von 0,5/h auf 0,8/h angehoben werden konnte, indem vom Arbeitgeber zusätzlich zum an der Wand befindlichen Händedesinfektionsmittelspender auch an der Narkosemaschine ein solcher montiert wurde (*p* = 0,01) [8]. In einer weiteren Studie von Koff et al. wurden die teilnehmenden AnästhesistInnen mit einem tragbaren Händedesinfektionsmittel(HDM)-Spender und einem Timer ausgestattet. In der Kontrollgruppe alarmierte der Timer in einem Intervall von 6 min, wenn keine HD von selbst durchgeführt wurde. Während des Tragens des Timers wurde die Frequenz der HD von 0,15/h auf 7,1/h (*p* = 0,008) für das behandelnde ärztliche Personal und von 0,38/h auf 8,3/h (*p* = 0,002) beim restlichen Personal erhöht. Mit dem Anstieg der Frequenz der HD war eine Reduktion der Kontamination des Anästhesiearbeitsplatzes und der peripheren Verweilkanülen (PVK) verbunden. Die „Healthcare-associated-infections“(HAI)-Rate wurde von 17,2 % auf 3,8 % reduziert (*p* = 0,02). Die Verwendung von tragbaren HDM-Spendern führte des Weiteren in einer anderen Studie zu einer Reduktion von ventilatorassoziierten Pneumonien auf der Intensivstation [9], wobei einschränkend beachtet werden muss, dass die Arbeitstechnik und -frequenz in einer Intensivstation nicht zwangsläufig mit der im OP vergleichbar sind. Eine Studie untersuchte das Durchführen einer Händedesinfektion in Abhängigkeit einer 60-sekündigen elektronischen Erinnerung alle 15 min, welche die Probanden dazu aufforderte, eine Händehygiene durchzuführen. Die Händehygiene wurde mit elektronischer Erinnerung im Median 2,1-mal, ohne Erinnerung im Median 0,2-mal durchgeführt (*p* = 0,006) [10]. Diese Ergebnisse werfen die Frage auf, ob beispielsweise eine Implementierung eines „Hygiene-Timers“ in bestimmte Software-Oberflächen (z. B. des PatientInnendaten-Management-Systems (PDMS)), welche auf Anästhesiearbeitsplätzen in Verwendung sind, sinnvoll wäre. Weiterführende Studien sollten allerdings klären, ob die beobachteten Effekte möglicherweise nur kurzfristig sind, oder ob Alarmierungen einen kontraproduktiven Effekt auf die Arbeitskonzentration haben könnten.

## Sollten wir bei der Atemwegssicherung 2 Paar Einmalhandschuhe tragen?

### Empfehlung.

Um das Kontaminationsrisiko der PatientInnenumgebung und des OP (insbesondere des Narkosearbeitsplatzes, des Anästhesiewagens oder der Computertastatur) zu reduzieren, empfehlen wir, die Narkoseeinleitung bis einschließlich Atemwegssicherung mit 2 Paar Einmalhandschuhen („double gloving“) durchzuführen. Unmittelbar nach Sicherung des Atemwegs mittels Tubus bzw. Larynxmaske wird empfohlen, das äußere Paar kontaminationsfrei auszuziehen und zu entsorgen, um eine Kontamination der Umgebung zu verhindern (bei dieser Gelegenheit kann ein Handschuh im Falle einer endotrachealen Intubation gleichzeitig zur sauberen Versorgung des Laryngoskops verwendet werden).

Anschließend sollte eine HD mit dem inneren Handschuhpaar durchführt werden. Generell können sichtbar saubere Handschuhe mit einem alkoholischen Händedesinfektionsmittel behandelt werden, sofern das Handschuhmaterial mit dem eingesetzten Händedesinfektionsmittel kompatibel ist und es der zeitliche Ablauf nicht anders zulässt [11].

### Diskussion.

Auch wenn vergleichsweise wenig Literatur existiert, in der eine vom Laryngoskop ausgehende Kontamination durch Mikroorgansimen des oberen Respirationstraktes des Anästhesie-Arbeitsplatzes beschrieben wird, gibt es doch einige Hinweise dafür, dass sich auf Laryngoskopen – sowohl auf den Spateln als auch auf den Griffen – eine Vielzahl potenziell pathogener Spuren sogar nach Aufbereitung wiederfinden [12]. Im Rahmen einer Studie wurde eine Kontaminationsrate von 57 % der Spatel und 86 % der Griffe der Laryngoskope detektiert, die bereits desinfiziert und aufbereitet (also bereit für den nächsten PatientInneneinsatz) waren [13]. Auch auf flexiblen fiberoptischen Laryngoskopen fanden Bhatt et al. Kontaminationen [14].

Die Hände der AnästhesistInnen kommen beim Atemwegsmanagement und der Endotrachealintubation unweigerlich mit Sekret der oberen Atemwege in Kontakt. Daraus resultiert bei mangelnder oder fehlender HD eine Kreuzkontamination des Anästhesiearbeitsbereichs [15–18]. Eine Literaturrecherche ergab 2 Studien, welche in einem simulierten Rahmen den Effekt des Double glovings beschreiben [15, 16]. In diesen wurde während der Einleitung der Narkose ein Fluoreszenzmarker benutzt und die Streuung desselbigen vom Atemweg der PatientInnen in den Anästhesiebereich ermittelt. Das Tragen doppelter Handschuhe während der Einleitungssequenz und das unmittelbare Verwerfen des äußeren Handschuhs nach dem Sichern des Atemwegs führte zu einer deutlichen Reduktion der Kontamination der PatientInnenumgebung. Diese Kontamination wurde weiter reduziert, wenn das Laryngoskop unmittelbar nach Benutzen mit einem Einmalhandschuh sauber verpackt wurde. Eine weitere Studie von Jaffe et al. überprüfte die Double-gloving-Technik in einem realen klinischen Szenario [19]. Darin wurde gezeigt, dass das Tragen zweier Paar Handschuhe zu einer signifikanten Reduktion (*p* < 0,01) von über 50 % der Kreuzkontaminationen führte.

Die Folgeschäden einer Kontamination des OP und seiner Umgebung durch Mikroorganismen aus dem Respirationstrakt von PatientInnen, welche möglicherweise rund um die Atemwegssicherung erfolgt, kann insbesondere in hygienischen Hochrisikobereichen (beispielsweise Gelenkimplantaten in der Orthopädie im Vergleich zu einer Hysteroskopie) unterschätzt werden. Die Durchführung einer Desinfektion der behandschuhten Hände, wie sie von den Autoren beispielsweise nach der Atemwegssicherung empfohlen wird, stellt aus hygienischer Sicht kein Problem dar [15, 16, 20]. Es ist derzeit nicht gängiger Bestandteil der klinischen Routine, eine HD mit angezogenen Latex- oder Nitrilhandschuhen durchzuführen. Das ist v. a. darin begründet, dass es keine eindeutige Datenlage zur Beständigkeit der Integrität der Handschuhbarriere unter dem Einfluss von alkoholbasierten Desinfektionsmitteln gibt. Pitten et al. untersuchten den Einfluss von 60 %igem (v/v) Isopropanol auf 4 unterschiedliche Einmaluntersuchungshandschuhe [21]. Obwohl sich Latexhandschuhe sogar besser desinfizieren ließen als die behandschuhte Hand, war die Integrität eines nitrilbasierten Untersuchungshandschuhs nach 30 s Desinfektion nicht mehr gegeben. Der Vollständigkeit halber sei erwähnt, dass diese Studie nicht alle erhältlichen Händedesinfektionsmittel und Handschuhmodelle einbezog und sich diese Arbeit v. a. auf Zugfestigkeit bzw. die Permeabilität des getesteten Materials bezog – für die Beständigkeit der Integrität mögen dies Indikatoren sein, mehr allerdings nicht. Des Weiteren beschreiben die Autoren, dass einige Handschuhmodelle nach mehrmaliger Administration von alkoholischem Händedesinfektionsmittel klebrig wurden [22].

Allerdings ist zu hinterfragen, ob der Handschuh je nach Prozessgestaltung dann nicht einfach ausgezogen werden kann, die Hände desinfiziert werden können und mit der unbehandschuhten Hand weitergearbeitet werden kann.

Die KRINKO (Empfehlung der Kommission für Krankenhaushygiene und Infektionsprävention (KRINKO) beim Robert Koch-Institut (RKI) [23]. Händehygiene in Einrichtungen des Gesundheitswesens Bundesgesundheitsbl. 2016; 59:1189–1220; 10.1007/s00103-016-2416-6) empfiehlt als Voraussetzung für die Desinfektion der behandschuhten Hände:

- Der Handschuh muss chemikalienbeständig gemäß EN 374 sein, wobei die Prüfung der sog. Durchbruchzeit von 30 min (Schutzindex der Klasse 2) mindestens einen Alkohol einschließen soll. Vom Hersteller der Handschuhe und vom Hersteller des Händedesinfektionsmittels darf es keine Angaben geben, die einer Desinfizierbarkeit des Handschuhs entgegenstehen.
- Der Handschuh soll nur während der Versorgung an ein und demselben Patienten verwendet werden und ist nach Beendigung der jeweiligen Tätigkeit abzulegen.
- Sofern es der Arbeitsablauf zulässt, sollte der Wechsel von Einmalhandschuhen parallel zu den Indikationen der Händedesinfektion erfolgen, d. h. immer dann, wenn die Indikation für eine Händedesinfektion gegeben ist, aber Handschuhe getragen werden. Im Ausnahmefall können behandschuhte Hände anstelle eines Handschuhwechsels desinfiziert werden, wenn andernfalls der Arbeitsablauf nicht gewährleistet werden kann, z. B. bei Tätigkeiten am selben Patienten aber zwischenzeitlichem Kontakt mit unterschiedlich kontaminierten Körperbereichen.
- Kriterium für die Entscheidung ist, dass der spezifische Arbeitsablauf keine Zeitspanne für die Lufttrocknung der desinfizierten Hände nach der Desinfektion vor dem Anlegen der neuen Handschuhe gewährt.

## Was sollten wir bei der i.v.-Injektion von Medikamenten beachten?

### Empfehlung.

Die i.v.-Applikation von Medikamenten kann ein Beitrag zur Entstehung von SSI, aber insbesondere auch katheterassoziierter Infektionen, sein. Deshalb ist ein besonders sorgfältiger und hygienegerechter Umgang mit Gefäßzugängen und Applikationen von Medikamenten erforderlich. Grundsätzlich sind Ports, über die Medikamente verabreicht werden, vor jedem Gebrauch zu desinfizieren. Je nach Dringlichkeit einer medikamentösen Applikation muss auch hier im Sinne des/der PatientIn gehandelt werden.

### Diskussion.

Die Anschlüsse einer PVK und 3‑Wege-Hähne werden in der intraoperativen Phase regelmäßig mit Bakterien kontaminiert. Diese bakteriellen Kontaminationen, welche sowohl gewöhnliche Bakterien der Hautflora (z. B. Mikrokokken oder Koagulase-negative Staphylokokken) als auch multiresistente Erreger (z. B. Methicillin-resistenter *Staphylococcus aureus*, Vancomycin-resistente Enterokokken oder multiresistente gramnegative Bakterien) umfassten, wurden in einer Studie auf mehr als 30 % der intraluminalen Oberflächen der getesteten 3‑Wege-Hähne, die im Rahmen von konventionellen Vollnarkosen benutzt wurden, festgestellt [24]. Im Folgenden möchten die Autoren durch einige ausgewählte Studien auf gewisse Schwierigkeiten im Umgang mit der i.v.-Injektion von Medikamenten hinweisen:

- Innerhalb des neuseeländischen Anästhesiepersonals war die Gesamt-Compliance, was die nationalen Empfehlungen für die Desinfektion der Injektionsanschlussstelle betrifft, gering: Nur 21 % der AnästhesistInnen in Neuseeland gaben an, eine Desinfektion des i.v.-Zugangs „immer“ oder „oft“ durchzuführen. Demgegenüber finden sich 32 % des Personals, die angeben, eine solche Maßnahme „nie“ durchzuführen [12]. Die Lage in Australien war noch prekärer: Dort gaben 40 % an, eine solche Desinfektion „nie“ durchzuführen [13]. Eine anonymisierte Datenerhebung einzelner Abteilungen könnte hier möglicherweise Verbesserungspotenzial aufzeigen und zur Qualitätssicherung beitragen.
- In einer prospektiven Beobachtungsstudie von 548 Patienten, die im Rahmen einer Operation eine Vollnarkose erhielten, fanden Loftus et al. heraus, dass 23 % aller 3‑Wege-Hähne intraoperativ kontaminiert wurden [14]. Für diese Kontaminationen waren öfter Bakterien verantwortlich, die auf dem „Adjustable-pressure-level*“*(APL)-Ventil des Narkosegerätes wiederzufinden waren, als jene Bakterien, die sich auf den Händen des Anästhesiepersonals oder des Oropharynx und der Axilla des Patienten wiederfanden. Die intraoperative Kontamination der 3‑Wege-Hähne war mit einer niedrigeren Rate an HD pro Stunde des Anästhesiepersonals und einer erhöhten 30-Tages-Mortalität assoziiert. Ein Einfluss auf die Frequenz nosokomialer Infektionen konnte nicht nachgewiesen werden. Die Art und Häufigkeit der Behandlung der 3‑Wege-Hähne sowie die Praxis der Medikamentenapplikation wurden in diesem Artikel jedoch nicht beschrieben.
- In einer prospektiven Studie über ambulante chirurgische Eingriffe waren die Kontaminationsraten von 3‑Wege-Hähnen nach einer 6‑stündigen Inkubationszeit ab Entfernung am Ende von Eingriffen ähnlich. In der Gruppe, in der Propofol verwendet wurde, betrug die Kontaminationsrate 17 %, in der ohne Propofolverabreichung 19 % [17]. Die Verfahren mit einer Propofolanästhesie waren länger (1–2 h gegenüber < 1 h) und mit einer größeren Anzahl verabreichter Medikamente und Anschlussinteraktionen verbunden als Verfahren ohne Propofol. Wenn die Probenentnahme aus dem Infusionsbesteck nach 24 h und 48 h Wartezeit wiederholt wurde, war ein Vorhandensein von sichtbarem Propofol in den Toträumen der Absperrhähne mit einem signifikanten Anstieg der Bakterienzahlen im Vergleich zum Infusionsbesteck ohne sichtbares Propofol oder zu Sets ohne Verwendung von Propofol assoziiert, was darauf hindeutet, dass selbst konservierungsmittelhaltiges Propofol das Bakterienwachstum in i.v.-Anschlüssen und Schläuchen in Verbindung mit längeren Verabreichungsdauern fördern kann. Die Autoren berichteten in diesem Artikel nicht über die Einhaltung einer Anschlussdesinfektion oder der intraoperativen HD.
- In einer prospektiven einfach-verblindeten Studie untersuchten Loftus et al. die Kontamination von gewöhnlichen 3‑Wege-Hähnen, die in einer Gruppe vor Gebrauch desinfiziert und in der anderen Gruppe nicht desinfiziert wurden [18]. In dieser Studie wurde beim Einbinden von 592 Operationen festgestellt, dass die bakterielle Kontaminationsrate durch Desinfektion der Anschlüsse der 3‑Wege-Hähne von 41 % auf 32 % reduziert werden konnte (*p* = 0,047; „adjusted odds ratio“ = 0,703). Dennoch war die Kontaminationsrate beider Gruppen hoch. Die identifizierten kontaminierenden Bakterien waren zu 52 % Koagulase-negative Bakterien sowie *Staphylococcus aureus* (1 %), *Pseudomonas aeruginosa* (1 %) und andere gramnegative Bakterien (1 %).
- In einer weiteren prospektiven Studie am selben Zentrum randomisierten Loftus et al. 468 Operationen und das zugehörige Anästhesiepersonal für eines von 3 Injektionsschemen [19]. (1) Ein geschlossener 3‑Wege-Hahn wurde mit Iso propanolhaltigem Desinfektionsmittel vor der Injektion desinfiziert, (2) dasselbe System ohne Desinfektion vor Gebrauch, (3) die gewöhnliche Praxis mit einem 3‑Wege-Hahn mit einem offenen Lumen. In der Gruppe (1) wurde ein auf 70 %igem Alkohol basierendes Desinfektionsmittel vorgeschrieben und eine Einwirkzeit von 30 s vor dem Gebrauch vorgesehen. Allerdings wurde nicht auf die Technik der Desinfektion und auch nicht auf die Art der Bereitstellung des Desinfektionsmittels geachtet. Nach der Einleitung der Narkose war die Kontaminationsrate der Gruppe (1) 0 %, während sie in Gruppe (2) 4 % und in Gruppe (3) 3,2 % betrug.

Zusammenfassend kann festgestellt werden, dass Injektionsanschlüsse und 3‑Wege-Hähne regelmäßig mit potenziell pathogenen Erregern kontaminiert werden. Eine niedrige Anzahl von HD, eine hohe Anzahl an Medikamentenapplikationen und eine hohe Anzahl an Interaktionen mit den Anschlüssen eines i.v.-Katheters resultieren in einer höheren Gefahr der Kontamination selbiger. Auch wenn die Fachliteratur keine direkte Evidenz für das klinische Outcome liefert, so existiert dennoch eine nennenswerte Evidenz dafür, dass das Desinfizieren von Katheter‑, Injektions- oder 3‑Wege-Hahn-Anschlüssen mit einem alkoholbasierten Desinfektionsmittel das Risiko von katheterassoziierten Bakteriämien reduzieren kann [21]. Die Autoren empfehlen eine Desinfektion des benutzten Injektionsanschlusses vor jeder Benutzung durchzuführen (s. KRINKO „Prävention von Infektionen, die von Gefäßkathetern ausgehen“, Teil 2, [22]).

## Sollten Stechampullen wischdesinfiziert werden?

### Empfehlung.

Stechmembranen und Ampullenhälse sollen vor Gebrauch bzw. dem Aufziehen der Medikamente desinfiziert werden.

### Diskussion.

Die meisten Stechmembranen von Infusionen sind ebenso wie Brechampullen lediglich sauber, aber nicht steril, weshalb es Teil der klinischen Routine werden soll, dass diese vor Gebrauch mit einem geeigneten antimikrobiellen Mittel (z. B. einem alkoholischen Präparat) desinfiziert werden, sofern der Hersteller nicht ausdrücklich Sterilität der Durchstechmembran im Herstellungsprozess gewährleistet [25]. Eine neuseeländische Studie beobachtete 10 Anästhesieteams während 20 simulierter Fälle: Keiner der AnästhesistInnen desinfizierte die Stechmembranen vor dem Aufziehen der zu verabreichenden Medikamente, und alle TeilnehmerInnen waren davon überzeugt, dass diese Prozedur gemäß den Hygienestandards erfolgte. In dieser Studie wurden Mikroorganismen in 5 von 38 Auffangbehältern (13 %), auf 6 von 17 Nadeln (35 %) und in 10 von 197 Spritzen (5 %) nachgewiesen [26, 27].

## Welche i.v.-Katheter sollten wir unter maximalen Barrieremaßnahmen anlegen?

### Empfehlung.

Alle zentralen Venenkatheter (ZVK) sowie axilläre und femorale arterielle Zugänge sollen unter maximalen Barrieremaßnahmen gelegt werden – diese umfassen mindestens das Tragen eines chirurgischen Mund-Nasen-Schutzes des Typs II, einer Kopfbedeckung, eines sterilen Mantels, steriler Handschuhe und das Verwenden einer großzügigen sterilen Abdeckung. Radiale arterielle Zugänge sollten zumindest mit einer Kopfbedeckung, einem chirurgischen Mund-Nasen-Schutz des Typs II, sterilen Handschuhen und einer kleineren sterilen Abdeckung (z. B. Lochtuch) angelegt werden. Diese und weitere Empfehlungen sind Tab. 1 zu entnehmen – diese stammt in einer abgeänderten Form von einer weiteren Übersichtsarbeit von Schulz-Stübner [28].

**Table 1:** 

Maßnahme	VWK	Arterie^a^ (peripher), „Single shot“ peripherer Nervenblock	ZVK/PAK, zentrale^a^ Arterien	Punktionstracheotomie	Epiduralkatheter/peripherer Nervenkatheter zu Analgesie, Spinalanästhesie	Bronchoskopie
Haut, Antisepsis^b^ (Alkoholbasiert; remanenter Wirkstoff kann, muss aber nicht enthalten sein)	+	+	+	+	+	Bronchoskopie-Adapter wischdesinfizieren
Sterile Handschuhe	−	+	+	+	+	+
Steriler Kittel	−	−	+	+	+^e^	(+)
Sterile Abdeckung	−	Kleines Lochtuch^c^	Großes Feld^c^	Großes Feld, einschließlich Instrumentenablage	Großes Lochtuch	Ggf. Lochtuch
Steriler Ultraschallschutz	(+)	+	+	+	+	N. z.
OP-Haube^d^	−	(+)	+	+	+	+
Chir. Mund-Nasen-Schutz^d^	−	(+)	+	+	+	Gesichtsschutz und Brille oder chir. MNS Typ IIR (mit Augenschutz)

*VWK* Venenverweilkanüle, *ZVK* zentraler Venenkatheter, *PAK* Pulmonalarterienkatheter

+ empfohlen, (+) erwägenswert/nach Bedarf, − nicht erforderlich, *N. z.* nicht zutreffend

^a^Bei „zentralen“ Arterien wie A. femoralis oder axillaris und langstreckigem Katheter wie ZVK

^b^Nach Herstellerangaben, Einwirkzeiten beachten; sofern davon ausgegangen werden kann, dass die Prozedur längere Zeit in Anspruch nehmen kann, sollte ein remanenter Wirkstoff in Erwägung gezogen werden

^c^Mindestens den potenziellen Radius der Bewegungen des Seldinger-Drahtes abdeckend und zur Ablage erforderlicher Materialien

^d^Wenn nicht ohnehin im OP-Bereich getragen

^e^Bei Single-shot-Spinalanästhesie ohne Katheteranlage verzichtbar

Wie bei allen anderen Standards müssen in Akutsituationen Risiko und Nutzen abgewogen werden. Sofern die Zeit es zulässt, empfehlen die Autoren das folgende Vorgehen und berufen sich bei ihren Thesen auf das „Compendium of Strategies to Prevent Bloodstream Infections in Acute Care Hospitals“ [21] und auf die 2011 veröffentlichte Richtlinie des „Healthcare Infection Control Practices Advisory Committee“ (HICPAC) [29], welche die folgenden adäquaten Hygienemaßnahmen für ZVK sowie axilläre und femorale arteriellen Zugänge festlegen: Jedes in das Legen oder Wechseln eines Katheters involvierte Personal soll laut HICPAC mindestens einen chirurgischen Mund-Nasen-Schutz des Typs II, eine Kopfbedeckung, einen sterilen Mantel und sterile Handschuhe tragen. Der Patient soll während der Prozedur mit einer großen (möglichst „full body“), sterilen Abdeckung bedeckt werden.

### Diskussion.

In der klinischen Praxis wird Prozesszeiten wie der Narkoseeinleitung, inklusive Legen einer arteriellen Kanüle oder eines ZVK, besondere Aufmerksamkeit geschenkt. Dieses Vorgehen ist sinnvoll und rational, allerdings sollte immer ein optimaler Hygienestandard eingehalten werden. Weiters sollte bedacht werden, dass sämtliche Katheter, die in Notfallsituationen angelegt werden, im Verlauf gewechselt werden sollten.

## Wie lange vor Gebrauch dürfen Infusionen angestochen werden?

### Empfehlung.

Grundsätzlich soll die Zeitspanne zwischen dem Anstechen einer Infusion und der tatsächlichen Verabreichung an den Patienten so gering als möglich (unmittelbar vorab) sein. Nichtsdestotrotz sind Akut- oder Notfallsituationen denkbar (z. B. Vorbereitungen für eine/einen angekündigte/n SchockraumpatientIn), die das Vorbereiten von Infusionen rechtfertigen – in diesem Fall ist auf die jeweiligen hausinternen Richtlinien zu achten. Für eine/n solche/n NotfallpatientIn vorbereitetes und nichtverwendetes Material sollte unmittelbar entsorgt und nicht für andere PatientInnen weiterverwendet werden. In der momentan vorliegenden Literatur finden sich keine definierten Zeitlimits, die zwischen Vorbereitung und Verabreichung maximal einzuhalten wären. Da aufgrund vieler zu berücksichtigender Parameter eine exakte Angabe eines maximalen Zeitlimits nach Anbruch nicht benennbar ist und auch fallweise nicht sinnvoll sein kann, empfiehlt die KRINKO hierzu im Einzelfall eine Regelung vom Krankenhaushygieniker oder der zuständigen Apotheke in Form einer Arbeitsanweisung festzuhalten (s. Robert Koch-Institut (RKI) „Anforderungen an die Hygiene bei Punktionen und Injektionen“ [30]).

### Diskussion.

Zu dieser Fragestellung existiert wenig Literatur. In einer Studie wurde festgestellt, dass innerhalb von 8 h nach dem Anstechen von insgesamt 80 Ringer-Lactat-Infusionen, die von Fachpersonal nach dem Durchführen einer hygienischen Händedesinfektion vorbereitet wurden, kein Bakterienwachstum nachweisbar war [31]. Allerdings lässt diese Studie keine Rückschlüsse darauf zu, ob dies auch für andere Infusionen in anderen Szenarien gültig ist.

## Zusammenfassung

Jede Kollegin und jeder Kollege soll sich situationsspezifisch überlegen, welche der laut Leitlinien zu treffenden Hygienemaßnahmen erfüllt werden können. Den Autoren ist bewusst, dass, je nach Dringlichkeit der PatientInnenversorgung, unterschiedliche Aspekte im Mittelpunkt stehen müssen, um möglichst im Sinne der PatientInnen zu handeln. Bestimmt sind Situationen denkbar, beispielsweise bei der Versorgung eines Polytraumas im Schockraum, in denen auch ein Handeln gemäß Hygienerichtlinien hinter die Stabilisierung des Kreislaufs gestellt werden muss. Um trotz großen Zeitdrucks in Akutsituationen dennoch möglichst hygienisch korrekt arbeiten zu können, haben wir in dieser Übersicht einige Ansätze, wie beispielsweise das Tragen von 2 Paar Einmalhandschuhen („double gloving“) zur Intubation, das Versorgen des benutzten Laryngoskops mit einem Einmalhandschuh oder die Desinfektion der behandschuhten Hände vorgestellt. Außerdem wurde diskutiert, dass einer hygienischen Handhabung der 3‑Wege-Hähne besondere Aufmerksamkeit gelten sollte und diese in den meisten Fällen, angelehnt an die Ergebnisse von Beobachtungsstudien, verbessert werden kann. Für einige dieser Ansätze existiert derzeit noch wenig wissenschaftliche Evidenz – diese zu schaffen, wäre von großem Interesse für unsere PatientInnen, Pflege und letztlich die behandelnden ÄrztInnen.

AnästhesistInnen sind es gewohnt, meist unmittelbares Feedback über die Qualität ihrer Arbeit zu bekommen – auch wenn dies bei Hygienemaßnahmen selten zutreffend und schwerer auf gewissenhafte Arbeit der AnästhesistInnen zurückzuführen ist, können wir die postoperative Lebensqualität unserer PatientInnen durch gezielte Hygienemaßnahmen erhöhen, indem wir möglichst hygienisch arbeiten und so die Wahrscheinlichkeit einer SSI senken. Die vorliegende Arbeit belegt jedenfalls eindeutig, dass einer optimalen perioperativen Hygiene ein wesentlicher Stellenwert zukommt. Nach Auffassung der Autoren ist es die Pflicht eines/einer jeden Anästhesisten/In, die gültigen Richtlinien zur Hygiene im OP zu kennen und bestmöglich im Sinne unserer PatientInnen umzusetzen – mit dem Ziel der Reduktion vermeidbarer Infektionen.

## References

[1] t. Eurosurveillance editorial, ECDC’s latest publications, Euro Surveill, 23 (2018) 1806282.

[2] Loftus RW, Koff MD, Birnbach DJ (2015). The dynamics and implications of bacterial transmission events arising from the anesthesia work area. Anesth Analg.

[3] WHO (2020). WHO 5 Moments of Hand Hygiene: (1) before touching a patient; (2) before clean/aseptic procedures; (3) after body fluid exposure/risk; (4) after touching a patient; and (5) after touching patient surroundings.

[4] Paulson DS, Fendler EJ, Dolan MJ, Williams RA (1999). A close look at alcohol gel as an antimicrobial sanitizing agent. Am J Infect Control.

[5] Munoz-Price LS, Birnbach DJ (2013). Hand hygiene and anesthesiology. Int Anesthesiol Clin.

[6] G.W.H. Organization (2009). WHO guidelines on hand hygiene in health care: first global patient safety challenge clean care is safer care.

[7] Biddle C, Shah J (2012). Quantification of anesthesia providers’ hand hygiene in a busy metropolitan operating room: what would Semmelweis think?. Am J Infect Control.

[8] Munoz-Price LS, Patel Z, Banks S, Arheart K, Eber S, Lubarsky DA, Birnbach DJ (2014). Randomized crossover study evaluating the effect of a hand sanitizer dispenser on the frequency of hand hygiene among anesthesiology staff in the operating room. Infect Control Hosp Epidemiol.

[9] Koff MD, Corwin HL, Beach ML, Surgenor SD, Loftus RW (2011). Reduction in ventilator associated pneumonia in a mixed intensive care unit after initiation of a novel hand hygiene program. J Crit Care.

[10] Rodriguez-Aldrete D, Sivanesan E, Banks S, Mavarez A, Arheart K, Eber S, Munoz-Price LS (2016). Recurrent visual electronic hand hygiene reminders in the anesthesia work area. Infect Control Hosp Epidemiol.

[11] Scheithauer S, Häfner H, Seef R, Seef S, Hilgers RD, Lemmen S (2016). Disinfection of gloves: feasible, but pay attention to the disinfectant/glove combination. J Hosp Infect.

[12] Ryan AJ, Webster CS, Merry AF, Grieve DJ (2006). A national survey of infection control practice by New Zealand anaesthetists. Anaesth Intensive Care Med.

[13] Reynolds H, Dulhunty J, Tower M, Taraporewalla K, Rickard C (2013). A snapshot of guideline compliance reveals room for improvement: a survey of peripheral arterial catheter practices in Australian operating theatres. J Adv Nurs.

[14] Loftus RW, Brown JR, Koff MD, Reddy S, Heard SO, Patel HM, Fernandez PG, Beach ML, Corwin HL, Jensen JT, Kispert D, Huysman B, Dodds TM, Ruoff KL, Yeager MP (2012). Multiple reservoirs contribute to intraoperative bacterial transmission. Anesth Analg.

[15] Birnbach DJ, Rosen LF, Fitzpatrick M, Carling P, Arheart KL, Munoz-Price LS (2015). A new approach to pathogen containment in the operating room: sheathing the laryngoscope after Intubation. Anesth Analg.

[16] Gadalla F, Fong J (1990). Improved infection control in the operating room. Anesthesiology.

[17] Cole DC, Baslanti TO, Gravenstein NL, Gravenstein N (2015). Leaving more than your fingerprint on the intravenous line: a prospective study on propofol anesthesia and implications of stopcock contamination. Anesth Analg.

[18] Loftus RW, Brindeiro BS, Kispert DP, Patel HM, Koff MD, Jensen JT, Dodds TM, Yeager MP, Ruoff KL, Gallagher JD, Beach ML, Brown JR (2012). Reduction in intraoperative bacterial contamination of peripheral intravenous tubing through the use of a passive catheter care system. Anesth Analg.

[19] Loftus RW, Patel HM, Huysman BC, Kispert DP, Koff MD, Gallagher JD, Jensen JT, Rowlands J, Reddy S, Dodds TM, Yeager MP, Ruoff KL, Surgenor SD, Brown JR (2012). Prevention of intravenous bacterial injection from health care provider hands: the importance of catheter design and handling. Anesth Analg.

[20] Birnbach DJ, Rosen LF, Fitzpatrick M, Carling P, Arheart KL, Munoz-Price LS (2015). Double gloves: a randomized trial to evaluate a simple strategy to reduce contamination in the operating room. Anesth Analg.

[21] Yokoe DS, Anderson DJ, Berenholtz SM, Calfee DP, Dubberke ER, Eilingson KD, Gerding DN, Haas JP, Kaye KS, Klompas M, Lo E, Marschall J, Mermel LA, Nicolle LE, Salgado CD, Bryant K, Classen D, Crist K, Deloney VM, Fishman NO, Foster N, Goldmann DA, Humphreys E, Jernigan JA, Padberg J, Perl TM, Podgorny K, Septimus EJ, VanAmringe M, Weaver T, Weinstein RA, Wise R, Maragakis LL (2016). A compendium of strategies to prevent healthcare-associated infections in acute care hospitals: 2014 updates. Infect Control Hosp Epidemiol.

[22] Bundesgesundheitsbl, Prävention von Infektionen, die von Gefäßkathetern ausgehenTeil 2 – Periphervenöse Verweilkanülen und arterielle KatheterEmpfehlung der Kommission für Krankenhaushygiene und Infektionsprävention (KRINKO) beim Robert Koch-Institut, 2017. 10.1007/s00103-016-2488-3 28091693

[23] Empfehlung der Kommission für Krankenhaushygiene und Infektionsprävention (KRINKO) beim Robert Koch-Institut (RKI). Händehygiene in Einrichtungen des Gesundheitswesens Bundesgesundheitsbl. 2016;59:1189–1220. 10.1007/s00103-016-2416-6 10.1007/s00103-016-2416-6 PMC7079999 27558147

[24] Loftus RW, Koff MD, Burchman CC, Schwartzman JD, Thorum V, Read ME, Wood TA, Beach ML (2008). Transmission of pathogenic bacterial organisms in the anesthesia work area. Anesthesiology.

[25] U.S.P.U.a.t.N. F. (NF), Pharmaceutical Compounding. Sterile Preparations (2008).

[26] Gargiulo DA, Sheridan J, Webster CS, Swift S, Torrie J, Weller J, Henderson K, Hannam J, Merry AF (2012). Anaesthetic drug administration as a potential contributor to healthcare-associated infections: a prospective simulation-based evaluation of aseptic techniques in the administration of anaesthetic drugs. BMJ Qual Saf.

[27] Hemingway C, Malhotra S, Almeida M, Azadian B, Yentis S (2007). The effect of alcohol swabs and filter straws on reducing contamination of glass ampoules used for neuraxial injections. Anaesthesia.

[28] Schulz-Stübner S (2013). Infektionsprävention durch das Anästhesieteam. Anaesthesist.

[29] O’Grady N, Alexander M, Burns L, Dellinger E, Garland J, Heard S, Lipsett P, Masur H, Mermel L, Pearson M, Raad I, Randolph A, Rupp M, Saint S (2011). Guidelines for the prevention of Intravascular catheter-related infections. Clin Infect Dis.

[30] Bundesgesundheitsbl, Anforderungen an die Hygiene bei Punktionen und Injektionen, 2010. 10.1007/s00103-011-1352-8 21887628

[31] Haas R (2017). No bacterial growth found in spiked intravenous fluids over an 8‑hour period. Am J Infect Control.

